# Preparing practitioner research students in accredited health disciplines for careers in academia

**DOI:** 10.15694/mep.2019.000187.1

**Published:** 2019-10-08

**Authors:** Meryl Pearce Churchill

**Affiliations:** 1 James Cook University

**Keywords:** Academic, Employment, Postgraduate, Professional Development.

## Abstract

This article was migrated. The article was marked as recommended.

**Introduction:**The reasons commonly cited by health professionals embarking on doctoral studies are an interest in learning how to conduct research for the purpose of improving health practice, and an interest in pursuing a career in academia-this paper focuses on the latter-though the two are not mutually exclusive. The study assessed the current availability, and the skills needed for entry-level academic positions in accredited health professions in Australia.

**Method:**A single point-in-time survey of academic positions (below Associate Professor) across 41 Australian universities was conducted in mid-2019.

**Discussion:**For aspiring academics,contemporary professional experience is sought in teaching positions in accredited health professions in Australia. Applicants need to be able to articulate how their research informs their practice and how their practice informs their teaching. The professional development training of experienced health practitioner research students’ needs to be fit-for-purpose for their career aspirations.

## Introduction

Research students from accredited health professions who have worked for some time and remain employed full-time in their profession while embarking on a research degree part-time differ from their counterparts in that they already possess industry-relevant skills ( Offerman, 2011). Likewise, for professionals outside of health “.. a research degree compliments an already successful professional career” ( Barnacle, 2002, p.14). In health, the reasons commonly cited by students enrolling in a research degree are an interest in learning how to conduct research for the purpose of improving health practices and health outcomes, or an interest in pursuing a career in academia-the focus of this paper is on the latter group (though they are not mutually exclusive). In Australia, over 50% of research students in health aspire to a career in academia ( Edwards, Bexley and Richardson, 2011), but the availability of academic positions does not match the demand. For this reason professional development training aimed at preparing research graduates for employment outside of academia is now a formal part of the research degree, along with training on how to conduct research ( Australian Technology Network of Universities and Nous Group, 2016; Bridgstock, 2009; Edwards *et al.*, 2011). However, for practicing health professionals set on pursuing an academic career it may be argued that the industry focus of professional development training does not meet their needs.With the mandatory coursework component already a contentious issue-mainly due to time constraints around completion periods-there is not scope to extend the professional development training ( Thouaille, 2017). Furthermore, because “a sizeable minority of students .. do not plan to pursue an academic career” ( Edwards *et al.*, 2011, p.92) expanding the training to include academia-relevant skills runs counter to the original purpose of professional development training-namely, a focus on employability outside of academia. This paper argues that more attention needs to be paid to preparing experienced practitioners completing a research degree for academic teaching positions.

### Aim

With a specific focus on medicine and health disciplines, the aim of this paper is to (a) assess the current availability of entry-level academic positions in Australia, (b) outline the skills required for entry-level academic positions, and (c) provide some insights into how to prepare health practitioners for academia.

## Methods

To address objective (a) a once off, point-in-time survey of research graduate academic positions that were available at 41 Australian universities in June 2019. The survey only included university-advertised, academic positions open to research graduates in possession of a Masters, PhD, had submitted their thesis, or were nearing completion. Positions from entry level Lecturer (Level A) up to Senior Lecturer/Senior Research Fellow (below Level D), part-time/full-time, continuing/fixed term, contracts >6 months duration, teaching/research/mixed roles, and hybrid clinician-teaching positions were included. The following exclusions were applied: casual or sessional roles; and in health-specific positions: health educator where the primary employer was a State health department or hospital, and where the positions were not advertised through a university. The proportion of health-related positions relative to all other disciplines was quantified. In three cases the following assumptions were applied: two advertisements mentioned that multiple positions were available without specifying the number-each case was conservatively counted as two positions; one position was offered as either a full-time position or two part-time positions-it was counted as one position.

To address objective (b), and for the purpose of identifying gaps in the professional development training of research students, the selection criteria listed in each of the above-mentioned health-related positions were examined to identify the skills commonly sought in entry-level academic positions.

## Results/Analysis

Of the 359 academic positions available in June 2019 across Australia, the largest proportion were in health (37.0%; n=133), followed by Science, Technology and Engineering which together comprised 34.5% (n=124) of positions ( Table 1). In health, 66.9% (n=89) were in accredited professions; the remaining 33.1% (n=44) were in non-accredited fields such as microbiology, biostatistics, and public health ( Table 2). Psychology, medicine and nursing positions dominated the accredited professions ( Table 2). Over half (50.6%) of the accredited professions were offered on a continuing basis, compared to 9.5% in non-accredited fields ( Table 3).

**Table 1.** Academic positions (below Associate Professor level; >6 months duration) at 41 Australian universities in June 2019 (n=359).

Discipline	n	%
Accredited health professions, non-accredited health	133	37.0
Science, Environmental Science, Agriculture	65	18.1
Computer Science, Information Technology, Data Management	36	10.0
Business, Finance, Governance, Politics, International Studies	35	9.7
Engineering	23	6.4
Social Sciences, Humanities	21	5.8
Education	11	3.1
Architecture, Surveying, Urban Planning	9	2.5
Music, Fine Arts, Performing Arts	9	2.5
Languages, English	9	2.5
Law	8	2.2

**Table 2.** Academic positions (below Associate Professor level; >6 months duration) in accredited health professions and other health-related fields at 41 Australian universities in June 2019.

Accredited health discipline	n
Psychology	19
Medicine	13
Nursing	11
Medical Radiation (various)	9
Physiotherapy	7
Neurosciences	6
Occupational Therapy	6
Veterinary	6
Dentistry-related	4
Pharmacy	3
Speech Pathology	2
Podiatry	1
Optometry	1
Chiropractor-related	1
Sub-total	**89**
**Non-accredited health fields**	
Molecular biosciences, microbiology	18
Biostatistics	9
Public health	6
Health economics	6
Biomedical engineering	3
Health communication, leadership, management	2
Sub-total	**44**

**Table 3.** Type of position (time allocation, and term of contract) in the 133 advertised practitioner and non-practitioner health-related roles (below Associate Professor level).

Type of position	Time allocation	Accredited health professions (%)	Non-accredited health fields (%)
Continuing	Full-time	24.1	2.3
	Part-time	6.0	0
	Applicants choice	4.5	0.7
Fixed term	Full-time	20.3	24.8
	Part-time	12.8	3.8
	Applicants choice	0.7	0
Practitioner: non-practitioner		**68.4**	31.6

The common selection criteria from the accredited health professions are summarised in Table 4. Apart from the entry-level positions, applicants were required to provide evidence of high levels of performance, demonstrated success, or a proven track record with expectations increasing with the level of the position and in accordance with the primary focus of the role (research-only; teaching-only). Terminology varied by institution.

**Table 4.** Common selection criteria in university-based, health positions in accredited fields (from entry-level to Senior Lecturer/ Senior Research Fellow) in Australia in June 2019.

Research-focused positions:
• Postgraduate degree/final stages/ post-doctoral experience and discipline-specific knowledge (additional criteria pertaining to: methodology, discipline-specific data handling, or subject knowledge were frequently listed as ‘desirable’ criteria).
• Work independently and in a team with working relationships ‘productive’ and ‘respectful’ across a range of disciplines and sectors (colleagues, administrative staff, students, external community and industry groups).
• Ability to develop new partnerships.
• Ability to meet deadlines, time management, organisational skills, manage multiple priorities.
• Oral and written communication skills.
• Ability to gain external funding, lead projects, develop and progress ideas into research outputs.
• High-level peer reviewed journal publications, lead the process or lead author.
• Abide by Workplace Health and Safety guidelines, university policies, and an understanding of ethical procedures.
**In addition to the above criteria, in positions involving teaching:**
• Be registered or eligible for registration with the appropriate Professional Board of Australia.
• Ability and ‘willingness’ to teach across all levels, and supervise research students.
• Evidence of scholarship of teaching and enhancing the student learning experience, and experience with diverse student learning needs.
• Demonstrate how ones professional practice informs innovative teaching, and how their profession-based research informs teaching.

## Discussion

### Academic positions in health

Globally, a perceived lack of academic jobs is a detractor to potential applicants to academia ( Bexley, James and Arkoudis, 2011; Roach and Sauermann, 2010; Waaijer, 2017). The results of this survey show that in Australia while there remains a shortfall to demand, in general, health fared better than other disciplines ( Table 1), and particularly in accredited fields where continuing (50.6%) and full-time (64.9%, with a further 7.6% optional) positions dominated. Continuing positions offer a greater level of job security. Fixed-term and part-time contracts are often negatively perceived ( Bexley *et al*., 2011; Waaijer *et al.,* 2017). Yet, contrary to these perceptions, the part-time nature of the accredited professional roles enables (or requires) the applicant to remain active in their practice ( Table 3) thereby providing the incumbents with the flexibility to pursue either an academic or practitioner pathway should their career aspirations change. Likewise the blended positions may partly offset some of the detractors of working in education (e.g. salary), while retaining the attractors of the health profession (e.g. contribution to the community) ( Edwards *et al.,* 2011; Waaijer, 2017); but are not without other pressures ( Morris and Stanwick, 2018; Sethi *et al.,* 2017).

### Postgraduates’ skills required for academic positions in health

Specific to the practitioner-academic positions were the requirement for the applicant to be registered (or eligible) for registration. With an increasing emphasis on work-integrated learning in undergraduate teaching ( Currey, Considine and Khaw, 2011; Eley and Wilkinson, 2015), practitioners with contemporary clinical experience and an aptitude for innovative teaching in the clinical environment are needed in academia. In this study some of the part-time positions required the incumbent to be active in their profession for the balance of time. Such a requirement may facilitate undergraduate student access to professional placements, and connectivity to an industry network ( Pitt and Mewburn, 2016). Similarly, for succession-given the aging academic workforce long removed from active health practice ( Bexley *et al*., 2011; Coates and Goedegebuure, 2012; Currey *et al.,* 2011; Eley and Wilkinson, 2015; Hugo and Morriss, 2010; Larkin and Neumann, 2012)-it is important to attract health practitioners into teaching, particularly those with contemporary health practice experience. Furthermore, registered practitioners are needed on the teaching staff of accredited health degrees to maintain course accreditation. From the perspective of a research postgraduate applying for an academic position their link to their active current practice is a key selling point.

### Insights into how to prepare practitioner research students for academia

In many European (Netherlands, Germany, Belgium, Switzerland) and Scandinavian universities doctoral candidates are employed by a university to complete a PhD with no more than 25% of their time allowed to be dedicated to university teaching. The employment frees students from needing to find financial remuneration outside of the university ( De Grande *et al*., 2014; Huisman, de Weert and Bartelse, 2002; Tekian, 2014; Waaijer, 2017). This model is not markedly different to an Australian student on a scholarship engaging in casual undergraduate teaching and likewise does not necessarily provide the doctoral students with any teaching-related skills. As 80% of doctoral graduates in the Netherlands find work as researchers, research skills rather than teaching-related skills are relevant to their employment ( Waaijer, 2017). In the UK, teaching fellowships and Academic Apprenticeships are highly regarded and sought after for the mentoring and tertiary pedagogic training they provide to aspiring academics ( Institute for Apprenticeships and Technical Education, 2019). It might be argued that such apprenticeships put overseas postgraduates in a more advantaged position than their Australian counterparts when applying for academic roles. In this study, a minority of the advertised teaching-focused positions required the incumbents to hold, or be willing to commit to, a formal qualification (e.g. postgraduate certificate) in tertiary education. Formal training in health professional education has been shown to improve medical educators’ self-efficacy in the scholarship of teaching ( Sethi *et al*., 2016); though some researchers debate the value of gaining such qualifications prior to applying for academic positions ( Pitt and Mewburn, 2016).

Given their benefits to universities, casual and sessional teaching roles are in ready supply. Notwithstanding the grievances articulated by casual university teaching assistants ( Bexley *et al*., 2011; Ryan *et al*., 2013; Coates and Goedegebuure, 2012; Muzaka, 2009), for research students, sessional teaching roles provide an income and an opportunity to test the waters of an academic position ( Barr and Wright, 2019). Engaging students in university roles while they are studying was found to reinforce their interest in pursuing an academic career ( Andriole *et al*., 2010; D’Alimonte, 2016; De Grande *et al*., 2014; Jacobsen *et al*., 2018). Remodelling casual teaching roles into academic internships or apprenticeships could be further explored.

While casual teaching roles provide students with an opportunity to gain teaching experience ( Barr and Wright, 2019), research shows that casual and new university teachers generally lack the skills necessary for their roles and would welcome training ( Barr and Wright, 2019; Bexley *et al*., 2011; Gardner and Jones, 2011; Muzaka, 2009). The selection criteria results of this study show that universities require applicants with demonstrated experience in effective teaching, curriculum design, and innovative teaching methods linked to contemporary practice. However, the skills needed for effective academic teaching such as curriculum design, research student supervision, marking, and innovative assessment, and the pedagogy of student learning are not covered in research training ( Pitt and Mewburn, 2016). While there is little scope to expand the doctoral professional development training, this paper suggests, along with others outside the field of health, that the professional development training needs to be better tailored to the career aspirations of research students regardless of their discipline ( Bridgstock, 2009; Coates and Goedegebuure, 2012; De Grande *et al*., 2014; Gilbert *et al*., 2004; Thouaille, 2017).

## Conclusion

While “.. there is no shortage of PhDs willing to work in academia ..” ( Waaijer, 2017, p.10), it might be argued that we are disadvantaging practitioner research students by not better supporting them to enter, enjoy and be effective teachers in academia. The professional development training of research students and graduate teaching assistants needs to be fit-for-purpose for their career aspirations. More needs to be done to assist them in developing, identifying, articulating and evaluating how their research informs their workplace practice and how their workplace practice could inform their teaching.

## Take Home Messages

• Health professionals possess industry-relevant skills but need academia-related skills.
• Aspiring academics need to articulate how their research informs their health practice and how their practice informs their teaching.
• Professional development training during postgraduate studies provides the scope to gain academia-relevant skills.
• Tertiary institutions should ensure that professional development training is fit-for-purpose to the academic career aspirations of health practitioners.

## Notes On Contributors

During her 25 years as an academic Associate Professor Meryl Pearce Churchill has nurtured a passion for enhancing the learning experience of tertiary students. Her current academic role in the discipline of health focuses on the vocational outcomes of postgraduate students. https://orcid.org/0000-0002-6417-1621

## Declarations

The author has declared that there are no conflicts of interest.

## Ethics Statement

No human or animal subjects were involved in the study.

## External Funding

This article has not had any External Funding
