# Supplementary material for: Preparing practitioner research students in accredited health disciplines for careers in academia
Source: MedEdPublish (2016). 2019 Oct 8;8:187. [Version 1] doi: 10.15694/mep.2019.000187.1 (PMC10712528; doi:10.15694/mep.2019.000187.1)
Supplement: Peer review history

# Reviewer report: Reviewer response for version 1

**DOI:** 10.21956/mep.19926.r30218

**Published:** 2019-10-21

**Authors:** Helena Filipe

**Affiliations:** 1 Hospital of the Armed Forces/PL-EMGFA

This review has been migrated. The reviewer awarded 5 stars out of 5 The Author selected a relevant and exciting topic to explore in the area of life long learning/ continuing professional development (CPD) considering merging fields as research, clinical practice and teaching.Advancing the state of the discipline and propelling scholarship has been associated with better acceptance of the teaching role by clinicians and with improvement in clinical performance.Nevertheless, most clinical educators haven’t received focused training in medical education and usually recognize a challenge to reconcile both identities, as Cantillon and associates describe in their recently published scoping review*. Depending on the setting, research, teaching and clinical practice can find an idiosyncratic support hierarchy. Nevertheless, teaching competency is or should be invariably acknowledged.What is regarded as a professional duty and an inherent responsibility, in times with wavering recognition of the complexity in which clinicians develop their practice including purposefully allocated time and financial resources, the opportunity of academic progression alongside with clinical practice looks reasonable and fair.This article is welcome to be discussed by both worlds: academia and clinical practice, with a bridging avenue for those wishing to pursue the goal of combining clinical practice, clinical or translational research, and teaching.The writing is fluid and clear and the elaboration on the results attained provides clear, thorough and useful insight on the subject fulfilling the third objective of the study.The abstract provides a clear summary of the study and the introduction sets the background in a concise and clear writing supported by consistent evidence.The tables illustrate well the results and guide the reader to the following sections. The discussion is elaborate and includes the contrast with overseas data as with other disciplines but health. It is well organized and addresses the three main questions defined at the outset of the study. Variables are wisely correlated to draw significant conclusions with a practical impact. Clinical practice experience is often a critical CPD teaching requirement. Academia experience, scholarship advancement and teaching skills development make tandem with clinical practice to increase effectiveness of medical education.Best acknowledgement to Prof. Churchill, for bringing up a relevant topic on lifelong learning quality and for sharing the Australian experience.*Cantillon P, Dornan T, De Grave W. Becoming a Clinical Teacher: Identity Formation in Context. Academic Medicine, Vol. 94, No. 10 / October 2019

Reviewer Expertise:

NA

No decision status is available

# Reviewer report: Reviewer response for version 1

**DOI:** 10.21956/mep.19926.r30220

**Published:** 2019-10-11

**Authors:** Irine Sakhelashvili

**Affiliations:** 1 European University

This review has been migrated. The reviewer awarded 5 stars out of 5 There was some interesting information about the Australian experience. I would say that, based on European experience (including my country, Georgia), I expected that in Australia, researchers in health, schools, including post-doctoral students, would also be required to be involved in the academic teaching process. For Georgian PhD students it's mandatory assist their supervisors in teaching during first two years of study, besides the research activity. This obligation helps the future researchers to gain the skills for further working with the students in higher education schools.In my personal opinion, the teaching experience should be equally mandatory for both the healthcare practitioners and the researchers. This is important for sharing existing knowledge and transferring skills to the younger generation, also for raising their interest in scientific and academic activities. This is probably the concern of Australian educational policy, but it would be nice if they shared the opinion of the author and encouraged healthcare practitioners and graduate students to participate in teaching activities. At least but not a last, additional skills - more job opportunities.Thanks to the author for an interesting article and for sharing the Australian experience.

Reviewer Expertise:

NA

No decision status is available

# Reviewer report: Reviewer response for version 1

**DOI:** 10.21956/mep.19926.r30219

**Published:** 2019-10-09

**Authors:** Mzwandile Mabhala

**Affiliations:** 1 University of Chester

This review has been migrated. The reviewer awarded 3 stars out of 5 This paper will be a useful reference for students in professional education courses and the professionals that are aspiring to become academics.

Reviewer Expertise:

NA

No decision status is available

# Reviewer report: Reviewer response for version 1

**DOI:** 10.21956/mep.19926.r30217

**Published:** 2019-10-09

**Authors:** Felix Silwimba

**Affiliations:** 1 University of Lusaka

This review has been migrated. The reviewer awarded 5 stars out of 5 honestly, I was not aware of the difference s. I now I'm. I have spent much of my time in clinical practice and healthcare management.

Reviewer Expertise:

NA

No decision status is available
